# Supplementary material for: Parvalbumin interneurons gate amygdala excitability and response to chronic stress via kainate receptor-driven tonic GABAB receptor-mediated inhibition
Source: Mol Psychiatry. 2025 Jun 28;30(11):5093–107. doi: 10.1038/s41380-025-03093-y (PMC12532722; doi:10.1038/s41380-025-03093-y)
Supplement: Supplementary file 1 — Supplementary Data [file 41380_2025_3093_MOESM1_ESM.pdf]

Supplementary Figure 1.

A

property	control n=17 (8 mice)	CRS n=19 (7 mice)	stat. test	P value
RMP (mV)	61.44 ± 1.473	57.10 ± 1.324	t-test, t=2.199, df=34	*p=0.0348
half-width (ms)	1.241 ± 0.1011	1.17 ± 0.06144	t-test, t=0.6109, df=34	p=0.5453
threshold (mV)	36.17 ± 1.090	36.11 ± 1.043	t-test, t=0.04231, df=34	p=0.9665
fAHP (mV)	10.61 ± 0.6937	11.39 ± 0.6186	t-test, t=0.8485, df=34	p=0.4021

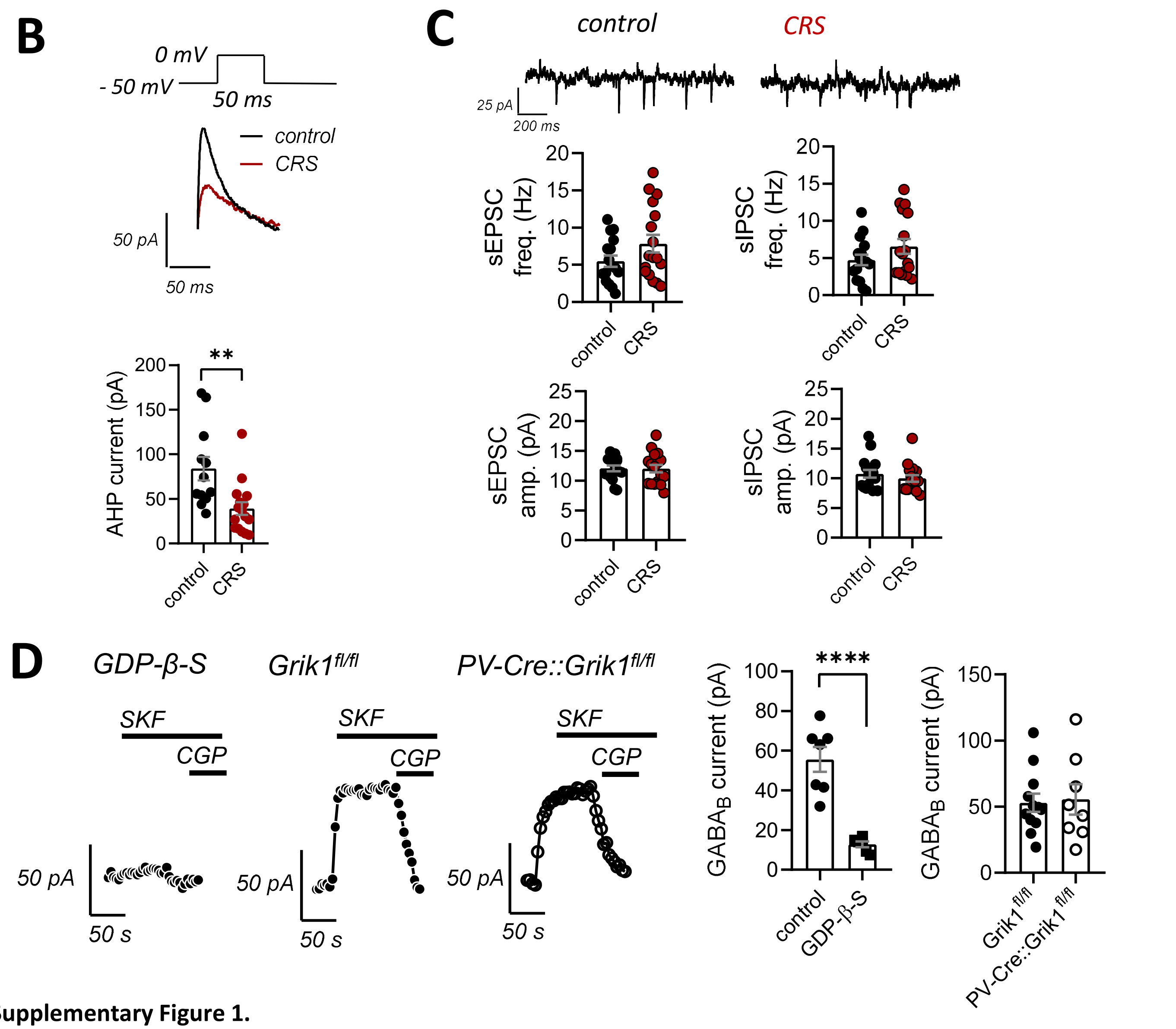

Supplementary Figure 1.

A. Membrane properties of LA principal neurons (PN) in control and CRS exposed animals. RMP – resting membrane potential, fAHP – fast afterhyperpolarizing potential.

B. Representative traces and averaged data on the medium duration afterhyperpolarizing (AHP) current amplitude, in the LA PNs of control and CRS exposed animals (control, n=12 (5 mice), CRS, n=16 (3 mice), Mann-Whitney test, U=29, **p=0.0012)

C. Example traces for recordings of spontaneous synaptic activity from LA PN neurons, using a low-chloride containing electrode filling solution at -50 mV holding potential. Under these conditions, sEPSCs and sIPSCs are observed as inward and outward currents, respectively. Pooled data on the sEPSC and sIPSC frequency and amplitude for control and CRS groups (control, n=16 (8 mice), CRS, n=17 (7 mice); sEPSC: t-test t=1.296, df=31, p= 0.205; sIPSC: t-test t=1.476, df=31, p= 0.15).

D. Outward currents in response to application of $GABA_B$ receptor agonist SKF97541 (25 µM) in LA PNs of C57/BL6 mice (control, n=7 (3 mice), GDP-β-S, n=6 (3 mice); t-test, t=6.108, df=11, ****p<0.0001), and $Grik1^{fl/fl}$ and PV-Cre::$Grik1^{fl/fl}$ mice ($Grik1^{fl/fl}$, n=12 (5 mice), PV-Cre::$Grik1^{fl/fl}$, n=8 (3 mice); t-test, t=0.2039, df=18, p= 0.8407). The G-protein inhibitor GDP-β-S (750 µM) was added to the pipette solution. All the recordings were done in the presence of 50 µM of DAP-5, 200 µM picrotoxin, 50 µM GYKI 53655. The $GABA_B$ receptor antagonist CGP55845 (5 µM) was added in the end of the experiment, and fully blocked the SKF97541 induced current.

All the data are presented as mean ± S.E.M.

Supplementary Data 2

A. Membrane properties of LA principal neurons in *Grik1*$^{fl/fl}$ and *PV-Cre*::*Grik1*$^{fl/fl}$ mice

genotype	Resting Vm (mV)	Rheobase (pA)	AP threshold (mV)	AP halfwidth (3rd AP, ms)
Grik1$^{fl/fl}$ (n=20)	-61.67±0.9743	93.68±3.763	-36.63±0.6859	1.265±0.07272
PV-Cre::*Grik1*$^{fl/fl}$ (n=20)	**-57.46±1.807* p=0.0470**	91.05±5.667 p=0.7011	-35.51±0.7425; p=0.2748	1.148±0.03750; p=0.1626

B. Membrane properties of LA PV interneurons in control and CRS exposed mice

treatment	Resting Vm (mV)	Rheobase (pA)	AP threshold (mV)	AP halfwidth (3rd AP, ms)
PV-Cre::TdTomato				
Control (n=20)	-65.32±1.750	78.75±7.088	-35.52±1.508	0.6489±0.05486
CRS (n=13)	-65.46±1.565; p=0.9543	**125±14.98** p=0.004**	-34.81±1.478; p=0.739	0.6453±0.04618; p=0.9593
PV-Cre::Grik1$^{fl/fl}$				
Control (n=12)	-66.00±3.871	99.17±24.45	-39.49±2.053	0.4400±0.02011
CRS (n=12)	-63.83±2.128; p=0.6286	103.3±15.34; p=0.8865	-36.69±0.9693; p=0.2310	0.4800±0.03908; p=0.3726

Supplementary Data 2. Averaged data on resting membrane potential (Vm) and properties of the action potentials (AP) (rheobase, threshold and halfwidth) for LA principal neurons in *Grik1*$^{fl/fl}$ and *PV-Cre*::*Grik1*$^{fl/fl}$ mice (A); and for LA PV interneurons in control and CRS exposed *PV-Cre::TdTomato* and *PV-Cre::Grik1*$^{fl/fl}$ mice (B). Statistics are calculated with unpaired t-test.

Supplementary Figure 3.

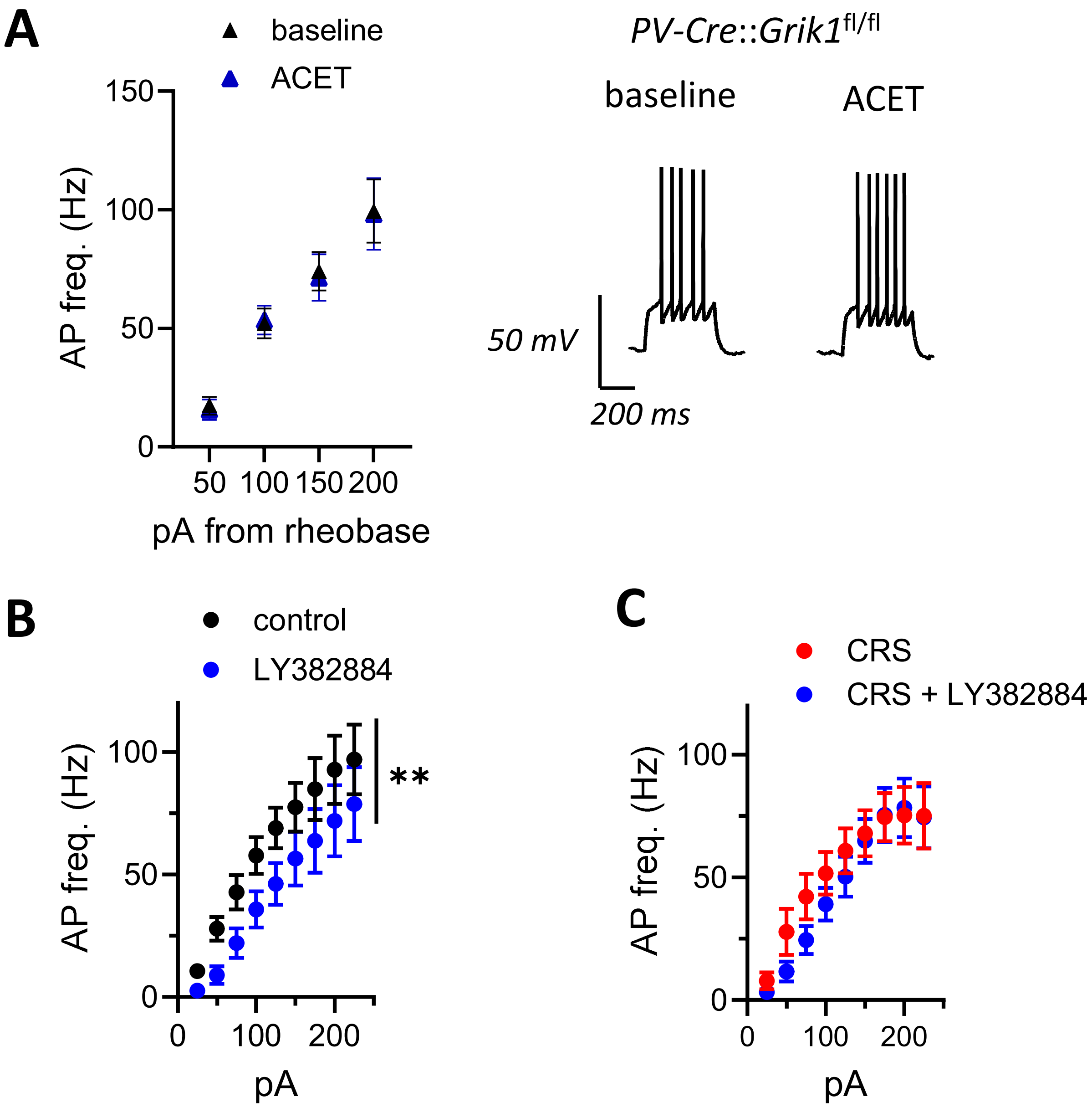

Supplementary Figure 3.

A. ACET (200 nM) has no effect on PV interneuron firing rate **i**n mice lacking GluK1 expression selectively in PV interneurons (*PV-Cre*::*Grik1*$^{fl/fl}$)(n=9 (4 mice). Example traces (50 pA current step) before and after ACET application.

B. LY382884 (10 μM) attenuates PV interneuron firing rate in response to depolarizing current steps in WT mice (n=10 (6 mice). RM ANOVA, $F_{(1, 9)}$ = 12.06, **p=0.0070)

C. The effect of LY382884 on PV interneuron firing rate is not observed in WT mice that have been exposed to CRS (n=7 (4 mice). RM ANOVA, $F_{(1, 7)}$ = 3.667, p=0.0971)

Supplementary Figure 4.

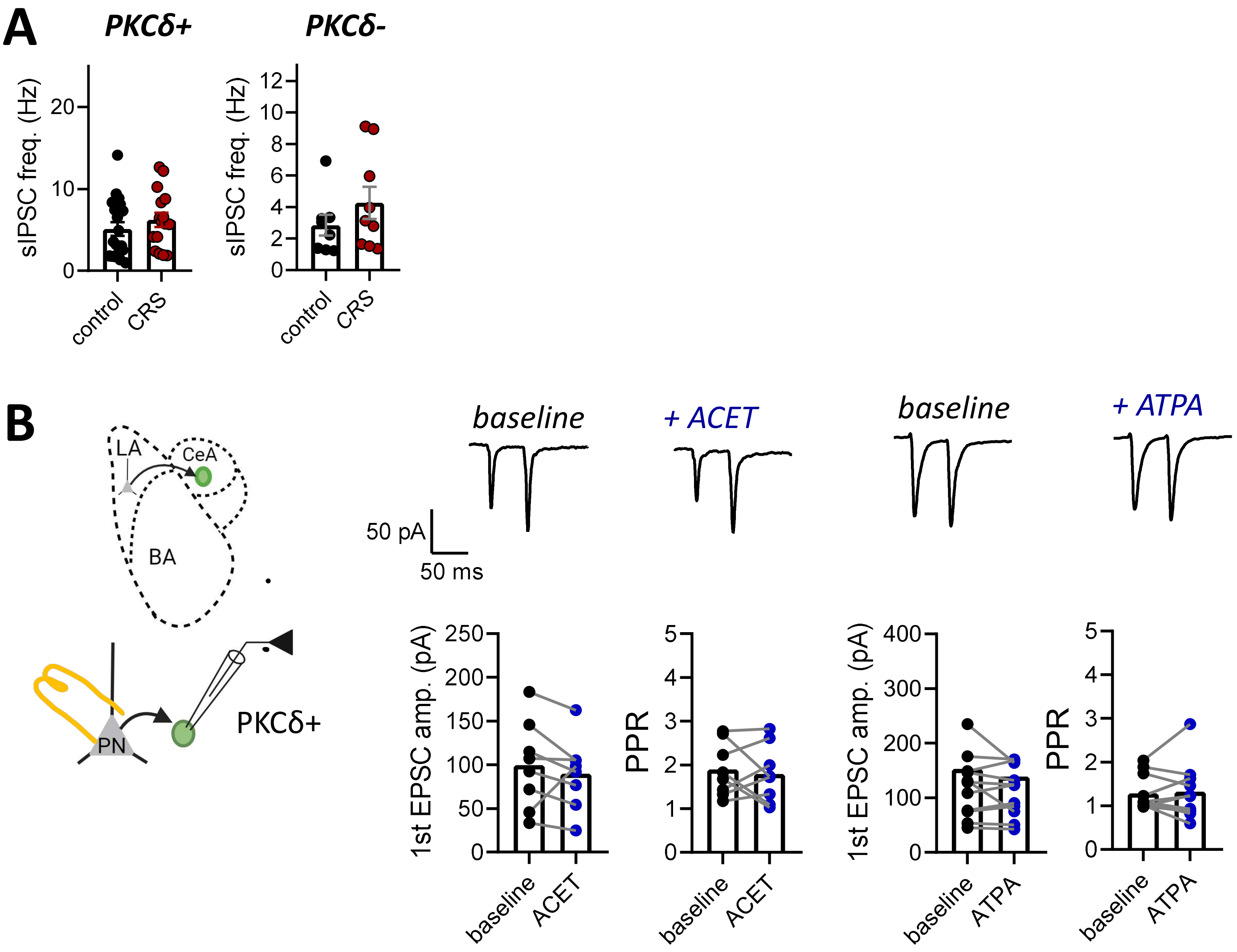

Supplementary Figure 4.

A. Pooled data on the sIPSC frequency , for the same recordings as shown in the Figure 5A (PKCδ+: control, n=19 (5 mice), CRS, n=16 (4 mice), t-test, t=0.9045, df=33, p=0.3723; PKCδ-: control, n=9 (4 mice), CRS, n=9 (5 mice), t-test, t=0.1988, df=16, p=0.8449).

B. Effect of ACET (200 nM) and ATPA (1 µM) on the amplitude and paired-pulse ratio of EPSCs in CeL PKCd+ neurons, evoked by stimulation of LA (1st EPSC amplitude: ACET: t=0.9624, df=7, p=0.3679; ATPA: t=1.503, df=11, p= 0.1610; PPR: ACET, n=8 (3 mice), paired t-test, t=0.4782, df=7, p= 0.6471; ATPA, n=12 (5 mice), paired t-test, t=0.3823, df=11 p=0.7095).

All the data are presented as mean ± S.E.M.
